# A comparison of different community models of antiretroviral therapy delivery with the standard of care among stable HIV+ patients: rationale and design of a non-inferiority cluster randomized trial, nested in the HPTN 071 (PopART) study

**DOI:** 10.1186/s13063-020-05010-w

**Published:** 2021-01-12

**Authors:** Mohammed Limbada, Chiti Bwalya, David Macleod, Sian Floyd, Ab Schaap, Vasty Situmbeko, Richard Hayes, Sarah Fidler, Helen Ayles, on behalf of the HPTN 071 (PopART) Study Team

**Affiliations:** 1 Zambart, University of Zambia, School of Medicine, Zambart House, Ridgeway Campus, Off Nationalist Road, P.O. Box 50697, Lusaka, Zambia; 2 Department of Infectious Disease Epidemiology, London School of Hygiene and Tropical Medicine, London, UK; 3 Imperial College and Imperial College NIHR BRC, London, UK; 4 Department of Clinical Research, London School of Hygiene and Tropical Medicine, London, UK

**Keywords:** HIV, Anti-retroviral therapy, Home-based ART delivery, Adherence clubs, Zambia

## Abstract

**Background:**

Following the World Health Organization’s (WHO) 2015 guidelines recommending initiation of antiretroviral therapy (ART) irrespective of CD4 count for all people living with HIV (PLHIV), many countries in sub-Saharan Africa have adopted this strategy to reach epidemic control. As the number of PLHIV on ART rises, maintenance of viral suppression on ART for over 90% of PLHIV remains a challenge to government health systems in resource-limited high HIV burden settings. Non facility-based antiretroviral therapy (ART) delivery for stable HIV+ patients may increase sustainable ART coverage in resource-limited settings. Within the HPTN 071 (PopART) trial, two models, home-based delivery (HBD) or adherence clubs (AC), were offered to assess whether they achieved similar viral load suppression (VLS) to standard of care (SoC). In this paper, we describe the trial design and discuss the methodological issues and challenges.

**Methods:**

A three-arm cluster randomized non-inferiority trial, nested in two urban HPTN 071 trial communities in Zambia, randomly allocated 104 zones to SoC (35), HBD (35), or AC (34). ART and adherence support were delivered 3-monthly at home (HBD), adherence clubs (AC), or clinic (SoC). Adult HIV+ patients defined as “stable” on ART were eligible for inclusion. The primary endpoint was the proportion of PLHIV with virological suppression (≤ 1000 copies HIV RNA/ml) at 12 months (± 3months) after study entry across all three arms. Viral load measurement was done at the routine government laboratories in accordance with national guidelines, annually. The study was powered to determine if either of the community-based interventions would yield a viral suppression rate drop compared to SoC of no more than 5% in its absolute value. Both community-based interventions were delivered by community HIV providers (CHiPs). An additional qualitative study using observations, interviews with PLHIV, and FGDs with community HIV providers was nested in this study to complement the quantitative data.

**Discussion:**

This trial was designed to provide rigorous randomized evidence of safety and efficacy of non-facility-based delivery of ART for stable PLHIV in high-burden resource-limited settings. This trial will inform policy regarding best practices and what is needed to strengthen scale-up of differentiated models of ART delivery in resource-limited settings.

**Trial registration:**

ClinicalTrials.gov NCT03025165. Registered on 19 January 2017

**Supplementary Information:**

The online version contains supplementary material available at 10.1186/s13063-020-05010-w.

## Background

Survival for people living with HIV (PLHIV) has dramatically improved with access to potent safe antiretroviral therapy (ART) [1]. However, to be effective, this treatment is currently delivered as daily oral tablets that require lifelong adherence [2]. Failure to adhere to treatment leads to viral recrudescence, clinical symptoms associated with immune dysfunction, increased risk of HIV transmission, and potential development of drug resistance [1].

The scaling up of antiretroviral therapy (ART) has been one of the most remarkable public health achievements in the last decade [3, 4]. There are an estimated 37 million PLHIV globally of whom 21 million are currently accessing ART. Sub-Saharan African countries home to approximately 19.4 million PLHIV have implemented an unprecedented scale up of ART especially in East and Southern Africa [3]. However, *how* to deliver sustainable, affordable ART to all PLHIV is an unanswered question that challenges global HIV care programs in high HIV burden limited resource settings.

Maintaining patients on ART requires a robust framework to monitor processes, outcomes, and long-term impact both at individual and programmatic levels. Retention on ART is a crucial indicator both at individual and programmatic levels and patient outcomes may be threatened if ART retention is poor or deteriorating [5–10]. Adherence to ART is necessary for individual patient outcomes as well as to reduce the risk of drug resistance from a public health perspective [11, 12]. Many studies have identified existing fragile health systems, inadequate human resources, transportation costs, frequent pharmacy pick-ups, and long waiting times at the clinic as significant barriers to ART retention in resource-limited settings [13–18] and without a change in the current model of ART delivery in resource-limited settings, lifelong ART for PLHIV may be unsustainable. As treatment coverage increases in the coming years, it is unlikely that human, financial, and physical resources will grow in proportion to this increase and there is therefore an urgent need to develop innovative models of ART delivery that can be implemented sustainably without compromising the quality of care.

Decentralization of HIV services to community level may be an important strategy to improve sustainability of programs [18]. Community models of ART delivery are one example of decentralizing HIV services from the health care facilities to the community. These models have been developed in various resource-limited settings and hold the promise of improving the continuum of care by decongesting the clinics and strengthening community engagement by linking community-based programs with the health care facilities [1, 19, 20].

Furthermore, the 2015 WHO guidelines [2] have recommended the provision of ART services in the community, but there is need for operational guidance and further evidence for this to happen in practice [1]. This resulted in the development of the “Differentiated Care” framework which has been defined as a client-centered approach that simplifies and adapts HIV services across the cascade, in ways that serve both the needs of PLHIV better and reduce unnecessary burdens on health systems [21].

The purpose of the framework is to provide guidance on how to address the barriers to treatment access and retention in care by optimizing models of care and drug delivery [21, 22] and focuses on stable patients who are defined as PLHIV adherent to treatment, who have no opportunistic infections and do not require frequent clinical consultations. However, this definition varies across different models dependent on access to resources, such as viral load monitoring [21].

## Evidence base of community models of ART delivery in resource-limited settings

Several studies have assessed the feasibility of community models of ART delivery in sub-Saharan Africa where the facility serves as the referral site, showing favorable outcomes in relation to retention in care and viral suppression [23, 24]. While 2 randomized trials have been conducted in Kenya and Uganda comparing home-based ART delivery to facility-based care [25–28], as well as observational studies on different models of ART delivery [23, 24, 29–33], most of these studies have been implemented in rural areas where patients live far from the health care facilities. We want to understand how such models would work in overcrowded, urban communities where community cohesion may be more limited. There is still a lack of evidence about which model is the most feasible and cost effective or whether patient outcomes will be as good as the current standard quality of care in high prevalence urban resource-limited settings. Therefore, additional data are required from alternate models of ART delivery that support long-term retention and virological suppression and evaluate which is the most feasible to fit into the current health system and community as well as the most-cost-effective strategy.

Our trial differs from the previous two trials described above in several aspects. Firstly, this will be the first study to rigorously evaluate two different models of ART delivery, home-based ART delivery and adherence clubs in a high HIV prevalence resource-limited urban setting in SSA compared to SoC for ART delivery. Secondly, this study uses a cluster-randomized trial design to do a non-inferiority comparison between SoC and each of the two models of community ART delivery. Finally, this trial will be able to assess the effect of shifting patients from routine ART care into the community and assess patient preferences and satisfaction unlike the trials conducted in Uganda and Kenya [25–28].

We designed a three-arm cluster-randomized non-inferiority trial comparing two different community model of ART delivery with the current standard of care to gather evidence on the impact of these models on patients’ clinical and virological outcomes, operational feasibility, acceptability, and cost-effectiveness to guide policy makers on best models to roll out in the context of universal treatment. An additional exploratory qualitative study was nested in this trial to complement the quantitative data. The reason for choosing this mixed-methods approach was to gather robust data to determine whether delivering ART and support outside the health care facility by community workers is safe and feasible and using viral suppression as our primary endpoint. In addition, several underlying social-contextual and health system factors such as delivering drugs outside the health care facility to patient homes or clubs, invasion of privacy, and community-based stigma needed to be explored. This design enabled us to robustly explore the safety, efficacy, acceptability, and feasibility of these models of ART delivery.

This paper looks at several aspects of our study design including why we chose a non-inferiority design, cluster versus individual randomization, as well as anticipated challenges, advantages, and disadvantages of the study design.

## Methods

### Study setting

This study was nested within the recently published HPTN 071 (PopART) trial and full details of this trial have been described elsewhere [34]. Briefly, HPTN 071 (PopART) was a cluster randomized trial done in 21 communities in Zambia and South Africa to estimate the effect of a combination HIV prevention package, which included door-to-door HIV testing services, linkage to care, immediate ART for HIV-positive individuals, and promotion of male circumcision for HIV-negative men, on HIV incidence between 2014 and 2018 [34]. Our nested study was conducted in the catchment population of 2 urban primary health care facilities that served two of the HPTN 071 trial communities in Lusaka, Zambia. The two sites were purposively selected for the following reasons:

1. Both communities were randomized to the intervention arms of the main HPTN 071 (PopART) trial, where community HIV care providers (CHiPs) were already employed to deliver the HIV combination prevention package through annual rounds of household visits throughout the entire communities.
2. Both communities have a high number of HIV patients in care (approximately 10,000) with a critical shortage of staff (0.8 per 1000 population) (http://www.aho.afro.who.int/profiles_information/index.php/Zambia:Health_workforce_-_The_Health_System) and are therefore an ideal setting to determine if these models of ART delivery benefit the health care facilities by decongesting the clinics and making treatment easier to access.

At the time of the study design (in 2016), the two communities had an HIV prevalence of approximately 20% among adults aged 18–44, and an estimated 70% of all PLHIV were accessing ART. This trial utilized existing CHiPs from the HPTN 071 trial to deliver the interventions. The CHiPs are trained members of the community, appointed to provide a package of basic services at household level, particularly HIV counseling and testing, screening for tuberculosis and sexually transmitted infections and linking household members for appropriate HIV prevention, treatment, and care services. The CHiPs work in pairs within allocated zones of the community (each zone consisting of 450–500 households), and this provided our study with the unique opportunity for them to provide the study intervention such as adherence support, symptom screening, and dispensation of pre-packed medications, while the CHiP zones offered a convenient and appropriate unit of randomization.

### Study design

This study was a three-arm cluster-randomized non-inferiority trial in a cohort of adult HIV+ patients on treatment in two urban health care facilities. Prior to the start of the main HPTN 071 (PopART) trial, mapping of the households and non-residential buildings was done, with a census to estimate the total number of adults and children allowing us to define a population of approximately 100,000 people per community to be included in the door-to-door intervention. This “intervention population” was then sub-divided into “CHiP zones” with each zone consisting of around 500 households and served by a pair of trained CHiPs. An additional qualitative study using observations of home delivery and clubs, interviews with PLHIV accessing ART via these models of delivery and group discussions with CHiPs delivering the intervention was nested within the main trial to complement the quantitative data.

### Randomization

The unit of randomization was a CHiP zone and random allocation of zones was done prior to recruitment of eligible participants. There were two communities with 104 zones in total: 54 in community 1 and 50 in community 2. Randomization was performed separately in the two communities. We restricted the randomization within each community on average values of key outcomes measured during the PopART intervention rounds to ensure balance across the three study arms on these factors. These were population size, HIV prevalence, proportion of PLHIV who attend the local clinic, and distance to the clinic.

For each community, one million potential permutations of allocations of zones into three trial arms (A, B, and C) were generated. Each permutation was assessed for balance across arms on the five factors, and if they were not within acceptable limits, then that permutation was discarded. From those remaining permutations that were balanced on the five factors, 10,000 were selected at random and numbered from 0000 to 9999. This provided 10,000 acceptable random allocations for each community with the final allocation to be selected from these at a public randomization ceremony.

We conducted two randomization ceremonies, one for each community separately on the 11 and 13 April 2017. In one community, we used a church hall and in the other community, a school hall. In each of the randomization ceremonies, we invited the CHiPs (108 and 100 in communities 1 and 2 respectively), 4 CHiP supervisors, 10 members of the PopART intervention team, 5 health care workers, 4 community advisory board members, 5 health care staff, and 1 community mobilizer to select the final allocation of zones to one of the three study arms.

We numbered 10 balls from 0 to 9 and asked 4 individuals from each of the above cadres to pick a ball, record the number, and put it back in the bag, giving a four-digit number between 0000 and 9999. This four-digit number was then used to select a single final allocation from the 10,000 generated earlier, allocating each zone to a trial arm: A, B, or C. Once this was done, we asked the CHiPs to take note under which arm their zones were allocated to and asked them to move towards their allocated arm in 3 separate corners of the room. A verification process was done by the study team to ensure that CHiPs serving their zones moved to the correct arm allocation. The next step included taking 3 sheets of papers, each labeled either HBD, AC, or SOC, folded, and put in a small box. From each of the 3 CHiP corners, we asked an individual (agreed by the CHiP teams) to pick 1 paper from the box. Once a paper was picked, it was revealed and the model of delivery was allocated to them. (i.e., if a CHiP from the allocated arm A picked a paper that was written HBD that was the model allocated to arm A, etc.).

### Study population and eligibility

All stable adult PLHIV (≥ 18 years) residing in the two urban communities enrolled in HIV care at the two primary health care facilities were eligible for study inclusion. Guided by the WHO classification for “stable” patients [22], we included all patients who were (1) on first-line therapy for at least 6 months, (2) virally suppressed using national guidelines [HIV RNA ≤ 1000 copies/ml] where viral load was taken less than 12 months prior to enrolment, and (3) had no other health conditions requiring the attention of a clinician. An additional eligibility criterion for our study included patients living within the study catchment area and being willing to provide written informed consent to participate in the study.

### Study procedures: screening and enrolment

During the enrolment period, the study staff screened all PLHIV attending the health care facility to determine who was stable according to the above definition. Stable patients were then asked whether they resided within the facility catchment area. Those who reported living in the catchment area were then met by the community mobilizer who confirmed their residence using the main trial intervention map. Having confirmed this, they were seen by the study nurse who was responsible for introducing the study to the potential participants and obtaining written informed consent. All participants were consented and enrolled before their random allocation was revealed to them. Participants who were allocated to the intervention arms (home-based delivery and adherence clubs) were given the choice of the allocated intervention or to continue receiving care in the clinic (SoC).

### Description of study interventions

#### A. Home-based ART delivery (HBD)

In zones randomized to HBD, a pair of CHiPs visited the participant in their homes once every 3 months to provide adherence support, symptom screening, and dispense pre-packed drugs. The participants were required to visit the clinic once every 6 months (twice in a year) for a routine clinical review and laboratory monitoring as per national guidelines. Table 1 gives a broad overview of the HBD model.

**Table 1:** Broad overview of the three ART delivery models

	Home-based delivery (HBD)	Adherence clubs (AC)	Standard of care (SoC)	Comments
	Clients are visited in their home by the CHiPs	Group of 20–30 clients meet at an agreed community venue led by a pair of CHiPs	Clients visit the clinics as scheduled	
**Number of visits/year**	2 HBD 2 clinic	2 clubs 2 clinic	4 clinic	
**ART dispensed**	3 months	3 months	3 months	
**Routine laboratory testing**	Every 12 months • Viral load • CD4 • Creatinine	Every 12 months • Viral load • CD4 • Creatinine	Every 12 months • Viral load • CD4 • Creatinine	As per routine national guidelines
**Frequency of clinical monitoring**	2 (every 6 months)	2 (every 6 months)	2 (every 6 months)	
**Process**	• Symptom screening • Adherence support • Health education and provision of condoms • Dispensation of pre-packed drugs	• Symptom screening • Adherence support • Group education and provision of condoms • Dispensation of pre-packed drugs	• Symptom screening • Adherence support • Health education and condom provision • Drug dispensation at pharmacy	

#### B. Adherence clubs (AC)

An adherence club consisted of a group of at least 20–30 stable PLHIV living within the same CHiPs zone and enrolled at the community health care facility. Each zone had one club and club members met once every 3 months at an agreed communal venue where they received adherence support, symptom screening, and pre-packed medications delivered by a CHiP pair. Club members were required to have 2 clinical visits (every 6 months) in a year for their routine clinical review and laboratory monitoring (Table 1).

In both the intervention arms, participants who developed any symptoms or became ill were referred to the health facility for further investigations and management. Participants found to have detectable viral loads, tuberculosis, and other common conditions were transitioned to clinic-based care for further follow-up.

#### C. Standard of care (SoC)—control arm

Participants living in zones allocated the SoC arm continued receiving care and ART prescriptions at the clinic. Currently, standard of care in Zambia includes patients visiting the clinic once every 3 months for drug collection and clinical monitoring depending on their last clinical and laboratory monitoring.

### Study hypothesis and rationale

The principal hypothesis is that clinical and virological outcomes in patients receiving the community-based interventions (HBD and AC) are non-inferior to those receiving care in the clinic (SoC) in an urban resource-limited setting. The rationale for the control arm selection is that care at the facility is the gold standard for stable ART patients in Zambia, and the rest of the world. The non-inferiority design applies to the primary outcome (proportion of participants who are virally suppressed), and the rationale for this design is that if viral suppression is found to be no worse in the intervention arms vs. the control arm, then the intervention will be preferable to the current standard of care. We set our non-inferiority margin at 5%, and the two primary comparisons will include a test of non-inferiority between home-based delivery vs. standard of care and adherence clubs vs. standard of care.

### Study endpoints and definitions

The primary endpoint in this trial was the proportion of patients with virological suppression at 12 months (± 3 months) after study entry across all three study arms. Viral load measurement used for our primary outcome was the measurement taken closest in time to 12-month post enrolment. If no measurement was taken within 90 days before or after this 12-month point, then the primary outcome was considered to be missing. We used the routine viral load testing results which according to the Zambian guidelines is defined as VL RNA ≤ 1000 copies/ml (based on the parameters of any assay performed through routine laboratory monitoring) and conducted at 6 and 12 months post ART initiation and thereafter annually for all stable patients. Secondary endpoints of this trial (assessed at the end of the trial) are as follows: (1) proportion of patients virally suppressed at 20 and 24 months after study entry (as measured by last VL taken between 20 and 24 months after study entry) and (2) proportion of patients loss-to follow-up (LTFU) and died 12 months after study entry. LTFU was defined as having no contact > 90 days after last missed scheduled appointment with unknown outcomes after study entry. Study participants who were transferred out of the health care facility were not considered LTFU but terminated from the study and other reasons for termination included death, LTFU, and study withdrawal; (3) proportion of patients retained in the intervention models after 12, 18, and 24 months; (4) clinical disease progression 12 and 24 months after study entry; and (5) qualitative research to assess the acceptability and functioning of the two models of ART delivery based on systematic structured observations of delivery, in-depth interviews, and focus group discussions from both the participants and provider (CHiPs and HCWs) perspectives.

In addition to the above outcomes, the study also looked at the performance, acceptability and feasibility of the two models of care using programmatic and routine health care facility data.

Retention on treatment during the study period was defined as a documented drug pick up in the last 120 days during the first 12 months after enrolment. Participants who shifted to another zone with a different intervention or shifted outside the study catchment area but continued to receive care at the facility were considered as retained in care. HIV disease progression was defined as proportion of participants who developed a new or recurrent WHO stage 3 or 4 condition at any given time after enrolment into study and death was defined at any point during the study due to any cause.

Additional process data were used to determine model retention, drug refills, and unscheduled or missed appointments after enrolment. For retention in the model of care, participants were considered non-retained if they transitioned back to standard of care or out of the study arms for any reason including co-morbidities, LTFU, death, participant opting out of the intervention, or withdrawal.

### Sample size and study power

Based on the data derived from the first annual round of the main trial, the number of adults who were known by the CHiP teams to be HIV+ and on ART at the time of the most recent follow-up visits averaged approximately 50 individuals per zone, with a harmonic mean of approximately 36 per zone. Assuming that 80% of such adults agree to participate in the study and have not moved out of the community within 12 months after enrolment, the number of study participants per zone who can contribute to the primary endpoint measurement will have a harmonic mean of approximately 30. Our study power calculations were done with an assumption that an average of 30 study participants per zone will contribute to the primary outcome measurement, and given there are 104 zones randomized to the 3 arms, this gives an estimated overall sample size of 3120 participants. We also assume that among the study participants in the “standard-of-care” arm, the percentage who are *not* virally suppressed 12 months after enrolment to the study is in the range 10–15%. The study was powered to determine if either of the community-based interventions would yield a viral suppression rate drop compared to SoC of no more than 5% of its absolute value.

To get the coefficient of variation *k*, formula *k* = *σ*/*π*. We assumed the lower end of our mean zone prevalence (to be conservative) so *π* = 10%, and we assumed that there would be approximately a 10% difference in the prevalence of not being virally suppressed between the lowest and highest prevalence zones (therefore ranging from 5 to 15%), equating roughly to a standard deviation (*σ*) of 2.5%. So, *k* = 2.5%/10% = 0.25. We also checked the power at a more conservative value of 0.3.

If the percentage of participants who are *not* virally suppressed at 12 months after study enrolment is 10% using a two-sided alpha value of 0.05 in the “standard-of-care” arm, and *k* = 0.25 or *k* = 0.3, study power is 93% and 91% respectively to show that a trial intervention arm is not inferior to “standard-of care.” The corresponding study power figures are 78% and 74% if the percentage of participants who are *not* virally suppressed at 12 months after study enrolment is 15% in the “standard-of-care arm.” The power was calculated using the formula for cluster-randomized non-inferiority trials by Hayes and Moulton [35]. So, the study, for our expected sample size, was estimated to have a power of between 74 and 93% under a range of different scenarios (Table 2). Our estimate of 10% of those not virally suppressed is consistent with research data from these two communities.

**Table 2:** Study power showing community ART provision is not inferior to standard-of-care, in terms of viral suppression 12 months after either enrolling into community ART or continuing with standard-of-care at the clinic

Standard of care, % not virally suppressed at 12 months after enrolment (%)	Non-inferiority margin (%)	Coefficient of variation *k*	Number of participants per cluster	Study power (%)
10	5	0.25	30	93
15	5	0.25	30	78
10	5	0.30	30	91
15	5	0.30	30	74

### Data collection and tools

This study was implemented by Zambart, a non-governmental research organization in Zambia recently having completed the HPTN 071 (PopART) trial and working closely with the Lusaka District Health Management Team and Implementing partners providing technical support to the health care facilities.

The study was based on prospectively collected routine data gathered for monitoring and evaluation purposes in the Zambian ART program. The study’s trained staff are collecting data from three sources: (1) clinic data where routine data is being collected at baseline and at every visit using the national health monitoring and information system (HMIS) and patient clinic records, (2) community Smartcare module specifically designed for the study where community interactions are recorded in a hand-held device and later synced with the national Smartcare database, and (3) study-related forms designed specifically for the study that will be used during enrolment and throughout the study period to measure the outcomes and processes of the study objectives. This will include consent forms, eligibility and enrolment forms, membership registers and attendance sheets, drug scripts, and study event forms.

To maximize the validity of this information, the study team worked closely with the health centers to improve the collection and management of these routine data. A CONSORT statement checklist has also been to improve the reporting of our RCT (Additional file 1) [36].

### Analysis plan

For the study outcomes, data analysis was conducted as for a non-inferiority cluster-randomized trial following the methods outlined by Hayes and Moulton [35]. We estimated the prevalence in each zone within each arm. The mean of the zone-specific values was then calculated for each arm along with its corresponding standard error and confidence interval (CI). Our non-inferiority margin was set at 5%. We then compared the control arm with each of the intervention arms using a one-sample *t* test to assess the evidence as to whether the mean difference between the control and intervention arms is less than 5%. If the upper limit of the 95% confidence interval (CI) for the difference is less than 5%, then we accept the intervention as being non-inferior. The primary analysis was unadjusted.

For our primary analysis, which is viral suppression at 12 months, we used intention to treat (ITT) analysis. Since participants in the intervention arm were offered the option to remain in care at the clinic, any who did so were included in the intervention model they were allocated to even though they did not select the allocated model of ART delivery. However, a potential concern with an ITT analysis is if the uptake of the alternative methods is low, or if participants move between study zones or arms resulting in a change to their model of delivery, then the intervention arms become similar to the SoC arm and we may bias the results towards equivalence. So, in addition to the ITT analysis, we performed a per-protocol analysis (PPA) comparing the outcomes in those who received SoC (i.e., participants who were allocated to the SoC arm and those from the intervention arms who chose to receive SoC) with those that received HBD/AC. This would help in interpreting the overall result and should be able to detect if there is, e.g., a much worse outcome in those opting for home delivery. We used PPA as a supportive analysis for the non-inferiority assessment. We also adjusted for potential confounding in the secondary analysis as the participants may no longer be balanced, as those who choose an alternative model may be different from those who do not, in some ways.

If a participant moved from an intervention zone to another zone which did not offer the intervention (or offered a different intervention) and therefore reverted to SoC, we decided to include them in the analysis in their original arm as per the principle of ITT. A potential disadvantage of this choice is that by *including* these individuals, we are making the intervention arm more similar to the SoC arm and therefore in a non-inferiority design this is less conservative. However, mobility between zones was only tracked in the two intervention arms, so if we chose to exclude individuals who we knew had moved zones, we would be removing more mobile individuals from the intervention arms but not from the SoC arm, and assuming that more mobile individuals are at greater risk of viral rebound then *excluding* those individuals would be less conservative as it could make the intervention arms look better. Weighing up these two options, it was thought that the latter issue introduced a greater risk of bias so the primary analysis would *include* those participants (while a sensitivity analysis excluding them would also be performed).

During the study period and towards the end of the study (2 months before the end of the intervention), a team of social scientists collected qualitative data from the two study sites. To ensure fair representation of the study sites, purposive sampling was used to select PLHIV from different age and gender groups as well as different areas of residence and socio-economic status. For staff, CHiPs delivering care through the two models were also selected to participate. Audio-recorded in-depth interviews (*n* = 24) were used to collect data from PLHIV and FGDs (*n* = 2) were used to collect data from CHiPs. Data were then triangulated methodically by longitudinal observations of delivery of the two models (*n* = 18). All audio-recorded IDIs and FGDs were then transcribed, and notes taken during observations expanded in office 2016 and analyzed using Atlas.ti 7.

### Ethical considerations

#### Approval

The study was granted ethical clearance from in-country authorities [University of Zambia Biomedical Research Ethics Committee (UNZABREC)], National Health Research Authority [NHRA], and the London School of Hygiene and Tropical Medicine ethics committee. The protocol had also been through regulatory review and approved by Division of AIDS (DAIDS) at NIH, who granted us permission to carry out this study as an ancillary study to the main trial and registered at ClinicalTrials.gov.

#### Consent

Written informed consent was obtained from all eligible participants and an information sheet about the study was provided to all participants by the research staff. Having signed the consent form, the research nurse then informed the participant which intervention they had been allocated to or whether they would continue to receive care at the clinic. Participants allocated to one of the two intervention arms were offered a choice of accepting the intervention model or to continue receiving care at the clinic. Participants who chose the intervention models could opt to receive care at the clinic at any point during the study period.

#### Participant safety and monitoring

Throughout the study, for all those participants who were receiving the interventions, study staff and CHiPs continuously assessed and monitored participant safety and ensured that participant confidentiality was maintained. Patients in the intervention arms who had symptoms requiring a clinician’s attention were referred to the research nurse at the clinic and those who were not present during the home or club visit were followed up by the CHiPs to determine if they did come to the clinic to pick up their drugs. The study also anticipated social harms and stigma as these could occur as a result of taking part in the study and participants might be treated unfairly or could have problems being accepted by their families or community members. Although such effects were expected to be minimal, the study staff and the CHiP teams monitored these closely throughout the duration of the study.

## Study implementation and challenges

### Randomization

**Fig. 1:**
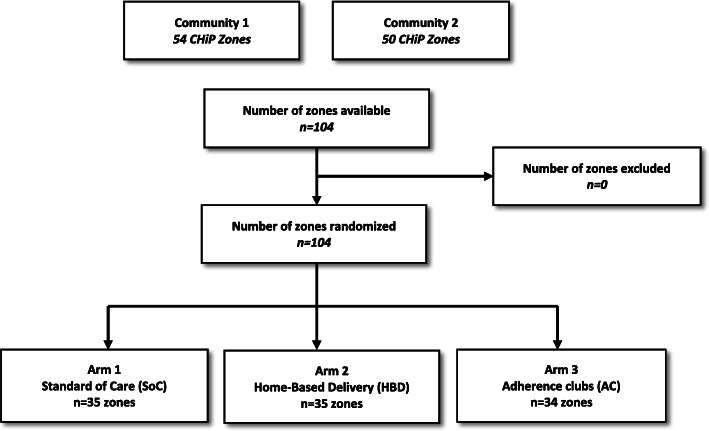
Randomization scheme

Randomization was conducted prior to the start of the study where statisticians provided 10,000 possible randomized allocations that met the restriction criteria, and a public randomization ceremony was held in both communities to select the final allocation of zones to the study arms. A total of 104 CHiP zones across both communities were randomized (35:35:34) to one of the three arms: (1) continue collecting ART at the clinic standard of care (SoC), (2) a choice of home-based ART delivery (HBD) or remaining in clinic-based care, or (3) a choice of being in an adherence club (AC) or remaining in clinic-based care (Fig. 1).

### Recruitment

**Fig. 2:**
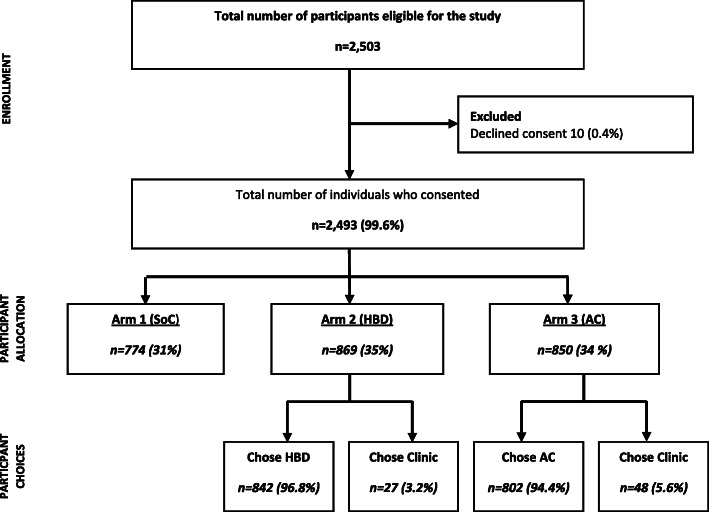
Recruitment and enrolment flow chart

All potential participants who fulfilled the definition of “stable patients on ART” in accordance with the pre-defined eligibility criteria during the screening process were sent to the research nurse for eligibility screening. Eligible participants who were able to demonstrate understanding of the study were asked to provide written informed consent. Having consented to the study, the participant’s residential address was located using the intervention map to identify the zone they were living in. Participants were then informed of the intervention arm they were allocated to. Participants allocated to the intervention arms had the option to take up the offer or continue receiving care at the clinic whereas those allocated to the control arm had no option but to continue care at the clinic. A total of 2503 stable patients were identified across the two communities between May and December 2017 who were eligible for inclusion in the trial and of these 2493 (99.6%) consented to participate and 10 (0.4%) declined consent (Fig. 2). Of the participants who consented, the majority were female (*n* = 1761, 71%). Median age of participants was 40 years (IQR 33–47) and the median years being on ART was 4 years (IQR 2–7).

### Challenges with recruitment

A total of approximately 9962 patients were screened across both communities between May and December 2017. We experienced a number of challenges during the screening process. First, most participants did not have a viral load test taken or had not received their results in the preceding 12 months as recorded in their clinical records at the time of screening. Thus, the study team had to send a patient for VL testing. Viral load results from the laboratory took between 1 and 3 months and study staff had to wait for another 1–3 months to determine eligibility. Secondly, some participants were not physically present at the clinic as they had their treatment supporters or “buddies” come and collect their drugs. The study team had asked the treatment supporters to inform the patient to come the following week or during their next scheduled visit. Thirdly, some patients were on treatment for less than 6 months and could only be enrolled in their consecutive visits if treatment duration was more than 6 months and they had a 6-month undetectable viral load result. Other reasons included having a detectable viral load, being on 2nd line treatment or having missed more than 2 clinical or drug pick up visit in the last 12 months.

Of the total number of patients who were considered stable, we further excluded a large number of patients as they were living either outside the study catchment area or in a community where the interventions were not offered.

## Discussion

This was one of the first studies conducted in an urban resource-limited high HIV burden setting that rigorously compared clinical and virological outcomes of patients participating in community models of ART delivery to current facility-based ART delivery as standard of care. In this paper, we present and describe the rationale for conducting a cluster-randomized non-inferiority trial to compare patient outcomes in community models of ART delivery among stable HIV+ patients in Zambia.

Most randomized trials are superiority trials and assess whether a new treatment is more efficacious than a placebo or current standard of care [37], whereas non-inferiority trials are intended to test whether a new treatment is no worse than a standard treatment by more than a specified margin and such trials have gained much attention in the last decade [37]. For our study, the rationale is to provide evidence on patient outcomes, acceptability, and feasibility of different models of ART delivery in resource-limited settings and whether these are novel strategies to scale up in the context of universal treatment in an effort to minimize the barriers to accessing care and treatment as we move towards the UNAIDS target of ending the epidemic by 2030. We used this design because in resource-limited settings, such as in Zambia, community models of ART delivery are being identified as a way of expanding treatment and the government through the National AIDS Council has engaged several in-country partners and researchers to pilot different models of ART delivery in order to generate information required to inform model standardization at national level for wider roll-out. Community models of ART delivery are likely to become part of standard of care if it does not negatively affect patient clinical outcomes as compared to the gold-standard which is the current standard of care.

The choice of this non-inferiority design was to test that clinical outcomes of patients under different models of ART delivery are not significantly inferior to the current standard of care, thereby showing that in resource-limited settings, these models of ART delivery can be considered as standard of care. Introducing these models of ART delivery as standard of care may potentially have long-term benefits such as decongesting the overburdened clinics to allow health care workers to concentrate on more complex patients, reduce patients’ financial and transport burdens of having to attend the clinics frequently for their drug pick-ups as well as improve community engagement and support towards HIV care and treatment. In contrast, a superiority trial design would not be feasible in resource-limited settings as it will require a lot of resources and would require health care workers to provide an intervention that shows superiority over the current gold standard of care.

This trial used cluster rather than individual randomization following in the footsteps of several large and ambitious trials of interventions against HIV and other infectious diseases in low and middle income countries that have helped guide health policy over the last decade or more [38]. These type of trials are used increasingly where delivery of intervention is at a group level and outcomes measured at patient level [39]. The decision to design a cluster randomized over an individually randomized trial for our study was that (1) it was ideally suited to study interventions that in practice had to be delivered at cluster (in this case, a zone) level, (2) it avoids the risk of contamination where participants from the control arm might receive some components of the intervention, and (3) this trial design was best designed to capture the effects of these interventions at community level.

In addition to the above, the communities were already divided into zones (clusters) by the main trial and it was logistically more feasible to train CHIPs on the particular interventions they would deliver rather than train them on all the interventions. It was also easier to control and monitor the interventions unlike individual randomization where it would have been difficult to deliver and monitor the interventions. In the case of adherence clubs, a club could be set up within each AC zone, meaning the clubs are close to participants’ homes, but if the trial was individually randomized, participants in the AC arm would be more geographically disparate and therefore in some cases far away from their allocated club, which could result in patients opting out of interventions.

This study has a robust design in being the first cluster randomized trial to explore outcomes of virological suppression, retention, feasibility, and acceptability of different ART delivery models and comparing it to the standard of care in a high prevalence urban setting and therefore provide us with evidence that could be generalizable to other sub-Saharan African settings and also inform policy regarding the best models to scale up. In addition to the above outcomes, this study also provided participants with a choice of continuing care at the clinic or receiving a community-based intervention and considered participant’s preferences towards the different models of ART delivery.

Despite the study strengths, non-inferiority trials have several challenges and limitations. As discussed in the analysis plan above, one of the challenges will be how best to analyze the data and whether to use ITT or PPA as we will have to deal with movements of participants between zones resulting in a change to their model of ART delivery. Assuming that both intervention arms are non-inferior to the standard of care arm, it would be desirable to determine whether one intervention arm is superior to the other (home delivery vs. adherence clubs). Although the study will not necessarily be powered to test this, other indexes on model uptake and retention, drop-out rates, and cost effectiveness can still be used to inform policy makers on model preferences.

Other limitations include the possibility of selection bias, where patients in the control arm may hear about the two models of delivery and may move from one zone to another which is providing the intervention. To avoid this, we asked patients at enrolment where they actually live and confirmed this with CHiPs who worked in those zones. Uniformity of implementing the interventions may change over time due to external factors such as bad weather and political climate. Another limitation was the substantial mobility and in-migration of participants within these urban communities as observed in the main trial [40, 41] thus requiring consideration of how to handle patients who relocate from one zone to another zone or community. There is also a source of bias as to who consents and who does not and those who take part in the study may not be representative of the general population. Another factor to be considered is that the study power might leave us underpowered if more than half of the adults in each zone opt to withdraw and return to standard of care as a result of stigma and disclosure. Other challenges included using routine data for measuring outcomes such as viral load results as most of these results were either missing or yet to be updated in the facility health care database and patient clinical records. To address this challenge, the study team worked closely with the clinic staff and laboratory staff to have viral load results entered in the clinic database and patient files.

As we move towards scale up of ART services to meet the UNAIDS target, there is need to provide evidence on the feasibility, outcomes, and cost effectiveness of differentiated care models and how best they can be combined alongside routine ART services. This trial will provide important data informing policy regarding best practices and what is needed to strengthen the scale up of differentiated care.

## Trial status

Enrolment into the trial commenced on 2 May 2017 and completed recruitment on 15 December 2017. The study recruited 2493 patients across the two urban communities and follow-up of participants ended in April 2019. The main trial outcome will be reported in 2020.

## Supplementary Information

**Additional file 1.**

## Data Availability

Further details on data collection tools and any data presented in this manuscript can be obtained from the corresponding author.

## Abbreviations

AC: Adherence clubs
AIDS: Acquired immune deficiency syndrome
ART: Antiretroviral therapy
CI: Confidence interval
HAART: Highly active antiretroviral therapy
HBD: Home-based delivery
HIV: Human immunodeficiency virus
HMIS: Health management information system
HPTN: HIV Prevention Trials Network
IAS: International AIDS Society
IQR: Interquartile range
ITT: Intention to treat
LTFU: Lost to follow-up
NHRA: National Health Research Authority
PLHIV: People living with HIV
PPA: Per protocol analysis
RNA: Ribonucleic acid
SOC: Standard of care
SSA: Sub-Saharan Africa
UNAIDS: Joint United Nations Programme on HIV/AIDS
VL: Viral load
VLS: Viral load suppression
WHO: World Health Organization

## References

[1] World Health Organization. March 2014 supplement to the 2013 consolidated guidelines on the use of antiretroviral drugs for treating and preventing HIV infection: recommendations for a public health approach. Geneva: World Health Organization; 2014. 24716260

[2] World Health Organization. Guideline on when to start antiretroviral therapy and on pre-exposure prophylaxis for HIV. 2015. http://www.who.int/hiv/pub/guidelines/earlyrelease-arv/en/. Accessed 13 Mar 2017. 26598776

[3] UNAIDS. Fact sheet - Latest statistics on the status of the AIDS epidemic. 2018. [Available from: http://www.unaids.org/en/resources/fact-sheet]. Accessed 4 Jan 2019.

[4] UNAIDS. Global AIDS Update. Geneva: UNAIDS; 2016. http://www.unaids.org/en/resources/documents/2016/Global-AIDS-update. Accessed 17 July 2018.

[5] Rosen S, Fox MP, Gill CJ. Patient Retention in Antiretroivral Therapy Programs in Sub-Saharan Africa: A Systematic Review. PLOS Medicine. 2007;4(10):e298. 10.1371/journal.pmed.0040298. 10.1371/journal.pmed.0040298 PMC2020494 17941716

[6] Tassie JM, Baijal P, Vitoria MA, Alisalad A, Crowley SP, Souteyrand Y. Trends in retention on antiretroviral therapy in national programs in low-income and middle-income countries. J Acquir Immune Defic Syndr (1999). 2010;54(4):437–41. 10.1097/QAI.0b013e3181d73e1b 20351559

[7] Assefa Y, Van Damme W, Haile Mariam D, Kloos H. Toward Universal Access to HIV Counseling and Testing and Antiretroviral Treatment in Ethiopia: Looking Beyond HIV Testing and ART Initiation. AIDS patient care STDs. 2010;24:521–5. 10.1089/apc.2009.0286 20672972

[8] Assefa Y, Jerene D, Lulseged S, Ooms G, Van Damme W. Rapid scale-up of antiretroviral treatment in Ethiopia: successes and system-wide effects. PLoS Med. 2009;6(4):e1000056. 10.1371/journal.pmed.1000056. 10.1371/journal.pmed.1000056 PMC2667265 19399154

[9] Assefa Y, Lynen L, Wouters E, Rasschaert F, Peeters K, Van Damme W (2014). How to improve patient retention in an antiretroviral treatment program in Ethiopia: a mixed-methods study. BMC Health Serv Res.

[10] Harries AD, Zachariah R, Lawn SD, Rosen S (2010). Strategies to improve patient retention on antiretroviral therapy in sub-Saharan Africa. Trop Med Int Health.

[11] Chammartin F, Zürcher K, Keiser O, Weigel R, Chu K, Kiragga AN, et al. Outcomes of patients lost to follow-up in African antiretroviral therapy programs: individual patient data meta-analysis. Clin Infect Dis. 2018;67(11):1643–52. 10.1093/cid/ciy347 PMC6233676 29889240

[12] Brennan AT, Maskew M, Sanne I, Fox MP (2010). The importance of clinic attendance in the first six months on antiretroviral treatment: a retrospective analysis at a large public sector HIV clinic in South Africa. J Int AIDS Soc.

[13] Hirschhorn LR, Oguda L, Fullem A, Dreesch N, Wilson P. Estimating health workforce needs for antiretroviral therapy in resource-limited settings. Hum Resour Health. 2006;4:1. 10.1186/1478-4491-4-1. 10.1186/1478-4491-4-1 PMC1402314 16438710

[14] Ware NC, Wyatt MA, Geng EH, Kaaya SF, Agbaji OO, Muyindike WR (2013). Toward an understanding of disengagement from HIV treatment and care in Sub-Saharan Africa: a qualitative study. PLoS Med.

[15] Tuller DM, Bangsberg DR, Senkungu J, Ware NC, Emenyonu N, Weiser SD (2010). Transportation costs impede sustained adherence and access to HAART in a clinic population in southwestern Uganda: a qualitative study. AIDS Behav.

[16] Hardon AP, Akurut D, Comoro C, Ekezie C, Irunde HF, Gerrits T (2007). Hunger, waiting time and transport costs: time to confront challenges to ART adherence in Africa. AIDS Care.

[17] Tomori C, Kennedy CE, Brahmbhatt H, Wagman JA, Mbwambo JK, Likindikoki S (2014). Barriers and facilitators of retention in HIV care and treatment services in Iringa, Tanzania: the importance of socioeconomic and sociocultural factors. AIDS Care.

[18] Fox MP, Rosen S. Patient retention in antiretroviral therapy programs up to three years on treatment in sub-Saharan Africa, 2007–2009: systematic review. Trop Med Int Health. 2010;15:1–15. 10.1111/j.1365-3156.2010.02508. 10.1111/j.1365-3156.2010.02508.x PMC2948795 20586956

[19] Fatti G, Grimwood A, Bock P (2010). Better antiretroviral therapy outcomes at primary healthcare facilities: an evaluation of three tiers of ART services in four South African provinces. PLoS One.

[20] Mills EJ, Nachega JB, Bangsberg DR, Singh S, Rachlis B, et al. Adherence to HAART: a systematic review of developed and developing nation patientreported barriers and facilitators. PLoS Med. 2006;3(11):e438. 10.1371/journal.pmed.0030438. 10.1371/journal.pmed.0030438 PMC1637123 17121449

[21] International AIDS Society (IAS) (2016). Differentiated care for HIV: a decision framework for antiretroviral therapy delivery: International AIDS Society.

[22] WHO (2016). Consolidated guidelines on the the use of antiretroviral drugs for treating and preventing HIV infection: recommendations for a public health approach.

[23] Tsondai PR, Wilkinson LS, Grimsrud A, Mdlalo PT, Ullauri A, Boulle A (2017). High rates of retention and viral suppression in the scale-up of antiretroviral therapy adherence clubs in Cape Town, South Africa. J Int AIDS Soc.

[24] Hagey JM, Li X, Barr-Walker J, Penner J, Kadima J, Oyaro P (2018). Differentiated HIV care in sub-Saharan Africa: a scoping review to inform antiretroviral therapy provision for stable HIV-infected individuals in Kenya. AIDS Care.

[25] Amuron B, Coutinho A, Grosskurth H, Nabiryo C, Birungi J, Namara G (2007). A cluster-randomised trial to compare home-based with health facility-based antiretroviral treatment in Uganda: study design and baseline findings. Open AIDS J.

[26] Jaffar S, Amuron B, Foster S (2009). Rates of virological failure in patients treated in a home-based versus a facility-based HIV-care model in Jinja, southeast Uganda: a cluster-randomized equivalence trial. Lancet.

[27] Kipp W, Konde-Lule J, Saunders LD, Alibhai A, Houston S, Rubaale T (2012). Antiretroviral treatment for HIV in rural Uganda: two-year treatment outcomes of a prospective health centre/community-based and hospital-based cohort. PLoS One.

[28] Kipp W, Konde-Lule J, Rubaale T, Okech-Ojony J, Alibhai A, Saunders D (2011). Comparing antiretroviral treatment outcomes between a prospective community-based and hospital-based cohort of HIV patients in rural Uganda. BMC Int Health Hum Rights.

[29] Decroo T, Rasschaert F, Telfer B, Remartinez D, Laga M, Ford N. Community-based antiretroviral therapy programs can overcome barriers to retention of patients and decongest health services in sub-Saharan Africa: a systematic review. Int Health. 2013;5(3):169–79. 10.1093/inthealth/iht016 24030268

[30] Bemelmans M, Baert S, Goemaere E, Wilkinson L, Vandendyck M, van Cutsem G, Silva C, Perry S, Szumilin E, Gerstenhaber R, Kalenga L, Biot M, Ford N. Community-supported models of care for people on HIV treatment in sub-Saharan Africa. Trop Med Int Health. 2014;19(8):968–77. 10.1111/tmi.12332. 10.1111/tmi.12332 24889337

[31] Wilkinson LS (2013). ART adherence clubs: a long-term retention strategy for clinically stable patients receiving antiretroviral therapy.

[32] Decroo T, Koole O, Remartinez D, dos Santos N, Dezembro S, Jofrisse M (2014). Four-year retention and risk factors for attrition among members of community ART groups in Tete, Mozambique. Trop Med Int Health.

[33] Luque-Fernandez MA, Van Cutsem G, Goemaere E, Hilderbrand K, Schomaker M, Mantangana N (2013). Effectiveness of patient adherence groups as a model of care for stable patients on antiretroviral therapy in Khayelitsha, Cape Town, South Africa. PloS one.

[34] Hayes R, Ayles H, Beyers N, Sabapathy K, Floyd S, Shanaube K (2014). HPTN 071 (PopART): rationale and design of a cluster-randomised trial of the population impact of an HIV combination prevention intervention including universal testing and treatment - a study protocol for a cluster randomised trial. Trials..

[35] Hayes RJ, Moulton LH. Cluster randomised trials, second edition. CRC Press; 2017. p. 398. 10.4324/9781315370286.

[36] Schulz KF, Altman DG, Moher D, the CG (2010). CONSORT 2010 Statement: updated guidelines for reporting parallel group randomised trials. BMC Med.

[37] Scott IA (2009). Non-inferiority trials: determining whether alternative treatments are good enough. Med J Aust.

[38] Hayes R (2018). Randomising towns to fight HIV. Significance..

[39] Elley C, Chondros P, Kerse N (2004). Randomised trials-cluster versus individual randomisation: Primary Care Alliance for Clinical Trials (PACT) network.

[40] Floyd S, Ayles H, Schaap A, Shanaube K, MacLeod D, Phiri M (2018). Towards 90-90: findings after two years of the HPTN 071 (PopART) cluster-randomized trial of a universal testing-and-treatment intervention in Zambia. PLoS One.

[41] Hoddinott G, Myburgh H, de Villiers L, Ndubani R, Mantantana J, Thomas A (2018). Households, fluidity, and HIV service delivery in Zambia and South Africa – an exploratory analysis of longitudinal qualitative data from the HPTN 071 (PopART) trial. J Int AIDS Soc.

